# Potential Therapeutic Agents for Feline Calicivirus Infection

**DOI:** 10.3390/v10080433

**Published:** 2018-08-16

**Authors:** Tulio M. Fumian, Daniel Enosi Tuipulotu, Natalie E. Netzler, Jennifer H. Lun, Alice G. Russo, Grace J. H. Yan, Peter A. White

**Affiliations:** 1 School of Biotechnology and Biomolecular Sciences, Faculty of Science, University of New South Wales, Sydney, NSW 2052, Australia; tuliomf@ioc.fiocruz.br (T.M.F.); d.enosi@unsw.edu.au (D.E.T.); natalienetzler@hotmail.com (N.E.N.); jennifer.lun@hotmail.com (J.H.L.); a.russo@unsw.edu.au (A.G.R.); grace.j.yan@unsw.edu.au (G.J.H.Y.); 2 Laboratório de Virologia Comparada e Ambiental, Instituto Oswaldo Cruz, FIOCRUZ, Rio de Janeiro 21040-900, Brazil

**Keywords:** feline calicivirus, antivirals, nucleoside analogues, non-nucleoside inhibitors, protease inhibitors

## Abstract

Feline calicivirus (FCV) is a major cause of upper respiratory tract disease in cats, with widespread distribution in the feline population. Recently, virulent systemic diseases caused by FCV infection has been associated with mortality rates up to 50%. Currently, there are no direct-acting antivirals approved for the treatment of FCV infection. Here, we tested 15 compounds from different antiviral classes against FCV using in vitro protein and cell culture assays. After the expression of FCV protease-polymerase protein, we established two in vitro assays to assess the inhibitory activity of compounds directly against the FCV protease or polymerase. Using this recombinant enzyme, we identified quercetagetin and PPNDS as inhibitors of FCV polymerase activity (IC_50_ values of 2.8 μM and 2.7 μM, respectively). We also demonstrate the inhibition of FCV protease activity by GC376 (IC_50_ of 18 µM). Using cell culture assays, PPNDS, quercetagetin and GC376 did not display antivirals effects, however, we identified nitazoxanide and 2′-C-methylcytidine (2CMC) as potent inhibitors of FCV replication, with EC_50_ values in the low micromolar range (0.6 μM and 2.5 μM, respectively). In conclusion, we established two in vitro assays that will accelerate the research for FCV antivirals and can be used for the high-throughput screening of direct-acting antivirals.

## 1. Introduction

Feline caliciviruses (FCV) are members of the *Caliciviridae* family (genus *Vesivirus*) and a major pathogen of cats worldwide. The virus has been associated with vesicular and upper respiratory tract disease, especially in multi-cat environments, such as shelters, colonies, and catteries, where FCV is detected in up to 40% of cats [1,2,3]. FCV infections typically cause a variety of clinical manifestations, such as acute respiratory disease and oral ulceration, with less common symptoms including pneumonia and acute arthritis/limping syndrome [4,5]. More recently, highly contagious virulent strains of FCV have emerged and were linked with severe disease (FCV-associated virulent systemic disease (VSD)) and high mortality rates (up to 50%) [6,7,8]. After the first description of FCV-VSD in 2000, outbreaks have occurred in the USA and Europe, which were associated with genetically distinct virulent FCV strains that have evolved locally [8,9,10,11,12,13]. The severe disease has a marked tropism for endothelial and epithelial cells of the skin and parenchymal organs and adult cats are often more severely affected than kittens [14,15].

The single-stranded, positive-sense FCV RNA genome (~7.7 kb) is VPg linked, polyadenylated, and includes three open reading frames (ORFs) [16]. ORF1 encodes non-structural proteins, including the 3C-like protease and 3D-like polymerase, ORF2 encodes the major capsid protein and ORF3 encodes a minor protein component of the virion [17]. Wei et al. [18] have demonstrated that the active form of the FCV polymerase is the bifunctional protease-polymerase (Pro-Pol) protein. FCV shows a high level of genetic diversity due to the lack of proofreading and the low fidelity of the viral polymerase, and genome recombination between different FCV strains during coinfections [4,19,20]. The phylogenetic classification of FCV strains, based on ORF2 nucleotide sequences, have demonstrated the circulation of a single genogroup (GI) around the globe, with the exception of Japan which has additional circulating strains that belong to a second genogroup (GII) [21,22,23].

Live-attenuated and inactivated vaccines against FCV have been available for over 40 years, using different FCV strains in monovalent or bivalent compositions [24]. Current vaccines do not prevent infection, viral shedding, or the development of FCV-VSD, but can reduce or even prevent clinical symptoms [2,7,10]. Moreover, there are no specific antivirals available against FCV infections and treatment options for FCV-VSD are only limited to supportive therapy.

Several studies have reported different antiviral strategies against FCV [25,26,27,28,29]. One of the earliest attempts used phosphorodiamidate morpholino oligomers (PMO) to treat cats during three FCV outbreaks (two caused by the FCV-VSD and one linked with a non-lethal FCV pathotype), showing promising results (79.6% vs. 9.7% of cats survived with or without PMO treatment, respectively) [27].

Mefloquine, a human-approved pharmaceutical compound used to prevent or treat malaria, demonstrated an antiviral activity against FCV in vitro at low micromolar concentrations (half maximal effective concentration, EC_50_ = 6.03 µM), although it demonstrated a poor selectivity index (SI = 3.7). The in vitro combination treatment with mefloquine and recombinant feline interferon-ω showed only a slight improvement of the IC_50_ [26].

Another class of compounds tested against FCV includes protease inhibitors (PIs). Synthetic PIs such as GC376 and NPI52 have demonstrated antiviral activity against FCV (EC_50_ = 35.2 µM and 0.02 µM, respectively). They were more effective against FCV when compared to the rhinovirus developmental PI rupintrivir (EC_50_ > 50 µM) and are considered broad-spectrum compounds showing additional activity against the feline coronavirus and human norovirus [29,30].

More recently, fexaramine, a synthetic agonist of the farnesoid X receptor, which plays a role in lipid metabolism, was shown to be effective at blocking FCV entry using in vitro assays, however, a single amino acid change (A539T) in the P2 domain of VP1 (the major capsid protein) was able to confer resistance [25]. In the same study, combination treatment with the aforementioned broad-spectrum PI (NPI52) showed synergistic antiviral activity (synergy log volume of 8.35 μM^2^%) and delayed the emergence of virus resistance.

Viral non-structural proteins, such as the protease and the RNA-dependent RNA polymerase (RdRp) are essential for viral replication, and therefore offer attractive antiviral targets. These enzymes lack host homologs, minimizing the chance of off-target effects and have been targeted successfully for the treatment of several viruses, including the human immunodeficiency virus (HIV) and hepatitis C virus (HCV) [31,32]. Using purified recombinant Pro-Pol, we established two in vitro assays for the screening of new FCV antiviral compounds. Representative nucleoside analogues (NAs), non-nucleoside inhibitors (NNIs), PIs, and the broad-spectrum nitazoxanide, were tested for FCV antiviral activity using both in vitro enzyme and cell culture-based methods. Here we identify two antiviral agents as potential therapeutic options for the FCV infection.

## 2. Materials and Methods

### 2.1. Cells and FCV

Crandell Rees Feline Kidney (CRFK) cells [American Type Culture Collection (ATCC) CCL-94)] were propagated in Eagle’s Minimum Essential Medium (EMEM, ATCC 30-2003) supplemented with 10% (*v/v*) fetal bovine serum (Sigma-Aldrich, St. Louis, MO, USA), 100 U/mL penicillin (Thermo Fisher, Waltham, MA, USA) and 100 μg/mL streptomycin (Thermo Fisher). Cells were grown at 37 °C with 5% CO_2_. The FCV strain F-9 (VR-782™; GenBank accession number M86379) was purchased from ATCC.

### 2.2. NNIs and NAs

Thirteen antiviral compounds were selected based on the reported in vitro antiviral effects against other caliciviruses [33] and included NNIs, NAs, PIs, and the broad-spectrum nitazoxanide. The following compounds were purchased from commercial vendors: JTK-109 (Dalton, Toronto, ON, Canada), TMC-647055 and Beclabuvir (BMS-791325; Taizhou Crene Biotechnology, Zhejiang, China), PPNDS (Molport, Riga, Latvia), quercetagetin, 2′-C-methylcytidine (2CMC), chymostatin and Famciclovir (Sigma-Aldrich), Compound 54 (School of Pharmacy and Pharmaceutical Sciences, Cardiff University, Cardiff, UK), Favipiravir (T705) and Sofosbuvir (MedChemExpress, Monmouth Junction, Middlesex County, NJ, USA), 7-Deaza-2-C-methyladenosine (7DMA; Carbosynth, Berkshite, United Kingdom), rupintrivir (In Vitro Technologies, Melbourne, VIC, Australia), GC376 (Focus Bioscience, Brisbane, QLD, Australia), and nitazoxanide (Sapphire Bioscience, Sydney, NSW, Australia). The stock solutions for all compounds were prepared in 100% dimethyl sulfoxide (DMSO) and aliquoted before storage at −20 °C.

### 2.3. FCV Pro-Pol Cloning, Expression and Purification

The Pro-Pol CDS from FCV Urbana (Genbank accession: L40021) was commercially synthesized in the pOA-RQ vector (Life Technologies, Carlsbad, CA, USA) and then sub-cloned into pET26b (Merck Millipore, Burlington, MA, USA) between *BamHI* and *SalI* restriction sites using forward and reverse primers: 5′-*AGGTAGGATCCAGTGGATTATAAAGACGATG*-3′ and 5′-*AGGTAGTCGACCACTTCAAACACATCAC*-3′ to produce pVRL345. For the expression of Pro-Pol containing a C-terminal histidine tag, pVRL345-transformed *Escherichia coli* BL21 (DE3) (NEB, Ipswich, MA, USA) were grown in Luria-Bertani media (2 L) at 37 °C with 100 μg/mL kanamycin until the OD_600_ was ~0.6. The culture was induced with 0.5 mM isopropyl β-d-1-thiogalactopyranoside (IPTG) for 20 h at 25 °C with shaking and bacteria pelleted by centrifugation. Chemical lysis of the pellet was performed as previously described [34], and lysates were loaded onto Ni^2+^ columns (BioRad, Hercules, CA, USA) and purified with an imidazole gradient (10–300 mM) using an AKTA start dual-buffer system (GE Healthcare, Little Chalfont, UK). The equilibration buffer consisted of 50 mM Tris-HCl, 500 mM NaCl, 10 mM imidazole, 5% glycerol (*v/v*) and 0.1% Triton X-100 (*v/v*), and the elution buffer was composed of the equilibration buffer with 300 mM imidazole. The purified protein was concentrated using an Amicon^®^ Ultra centrifugal filter (10 kDa cut-off, Millipore, Tokyo, Japan) and dialyzed against three buffers with decreasing NaCl concentration (300, 150, or 50 mM NaCl, with 25 mM Tris-HCl, 20% glycerol (*v/v*); 0.05% Triton X-100 [*v/v*]). All buffers were prepared at pH 8. The protein concentration was determined using a BCA Protein assay kit (Life Technologies).

### 2.4. Cytotoxicity Study

CRFK cells (3.5 × 10^4^ cells/well, 100 μL/well), were seeded into flat-bottom 96-well plates and incubated overnight at 37 °C. The cell monolayers were then treated with increasing concentrations of compounds in triplicate (0.2 µM–100 µM), followed by 48 h in incubation. DMSO (vehicle only, 0.5% (*v/v*)) was used as a negative control. The cytotoxicity of each compound was measured using the CellTitre-Blue viability assay kit (Promega, Madison, WI, USA) according to the manufacturers’ instructions. Fluorescence was measured on a FluoStar Optima microplate reader (BMG Labtech, Ortenberg, Germany) and the half maximal cytotoxic concentrations (CC_50_) were determined with GraphPad Prism v.7 (La Jolla, CA, USA).

### 2.5. Antiviral Screening Using Fluroescent RdRp Assays

RdRp activity was measured by monitoring the formation of double-stranded RNA (dsRNA) from a single-stranded RNA polycytidine template (poly(C)), as described previously [35,36]. RdRp activity was first optimized by increasing concentrations of enzyme (250–1000 ng per reaction), and varying sodium chloride (NaCl) concentrations (3–500 mM). Heat-inactivated RdRp was used as the negative control. Accumulation of the dsRNA product was measured using the fluorescent dye PicoGreen (Life Technologies). Reactions (25 µL) were performed in black-bottomed 384-well plates containing 600 ng of FCV Pro-Pol, 20 mM Tris-HCl (pH 7.5), 0.2 mM rGTP, 250 ng of poly(C) RNA, 5 mM MnCl_2_, 5 mM dithiothreitol (DTT), 0.005% Tween 20 (*v/v*) and 0.01% bovine serum albumin (BSA) (*v/v*). For antiviral screening, FCV Pro-Pol (10 µL) was incubated with 5 µL of each test compound (10 µM final concentration in 0.5% DMSO) or vehicle (0.5% DMSO) for 10 min at 30 °C before the addition of 10 µL of the reaction mixture with a further incubation of 15 min at 30 °C. Reactions were terminated with 10 mM EDTA, followed by incubation with PicoGreen and dsRNA quantitation [35,36]. GraphPad Prism v.7 was used to plot the half maximal inhibitory concentration (IC_50_) values.

### 2.6. Protease FRET Activity Assay and Antiviral Screening

The amino acid (aa) sequence of the cleavage site between the precursor leader capsid (LC) and the mature capsid protein (VP1) of the FCV genome was synthesized as a fluorogenic substrate peptide Dabcyl-FRLE↓ADDG-Edans (GenScript, Piscataway, NJ, USA) and a stock solution (10 mM) was prepared in 100% DMSO. Protease assays were performed in 384-well plates using a reaction volume of 50 µL containing 50 mM HEPES, pH 7.5, 6 mM DTT, 0.5 mM EDTA, 50% glycerol (*v/v*), 600 ng of FCV Pro-Pol and the fluorogenic substrate. The initial measurements to determine the Michaelis-Menten constant (*K_m_*) of the substrate were performed using increasing concentrations (0–100 µM) with incubation for 1 h at 37 °C. The influence of increasing NaCl concentration (3–130 mM) on protease activity as also evaluated. Following the determination of the *K_m_*, inhibition assays were performed with 50 µM of a substrate with either a PI (0–50 µM) or the vehicle control (0.5% DMSO) with incubation for 30 min at 37 °C. Upon cleavage of the substrate at the site indicated (↓), the quenching of Dabcyl fluorescence by the Edans group is abolished and the fluorescence generated was quantified at an excitation wavelength of 360 nm and an emission of 460 nm on a POLARstar plate reader. IC_50_ and *K_m_* values were determined using GraphPad Prism v.7.

### 2.7. Inhibition of FCV Plaque Formation in Cell Culture

FCV plaque reduction assays were performed as previously described [36,37]. CRFK monolayers (8 × 10^5^ cells/well) in 6-well plates were infected with approximately 80 plaque forming units (pfu) of FCV for 1 h at 37 °C, followed by the addition of semisolid agarose overlays containing different concentrations of compounds. Plates were incubated for 24 h, fixed and stained with crystal violet. Plaque numbers were determined for each drug treatment and the DMSO vehicle control was defined as maximal viral infectivity. To determine whether the combination of nitazoxanide and 2CMC had synergistic, antagonistic or additive effects, the percentage of inhibition of FCV infection was assessed over a dose-response matrix that included four concentrations of nitazoxanide (ranging from 0 to 0.6 μM) and 2CMC (0 to 4 μM). The effects of drug combination were assessed using SynergyFinder [38] and the zero-interaction potency (ZIP) model [39] was used to generate synergy scores from a dose-response matrix. Synergistic or antagonistic effects are shown as peaks above or below the horizontal plane, respectively. At least two independent experiments with triplicate datasets were performed for each treatment, with results presented as the mean with standard error of the mean (SEM).

### 2.8. FCV Genome Reduction Assay Using Reverse Transcription Quantitative Polymerase Chain Reaction (RT-qPCR)

RT-qPCR was used to evaluate the reduction in FCV RNA following antiviral treatment. Briefly, CRFK cells (2 × 10^5^ cells/well) in 24-well plates were infected with FCV at the multiplicity of infection (MOI) of 0.0005 for 1 h. Media was then replaced with media containing drug and incubated for a further 24 h. FCV viral RNA was extracted from the cells and supernatant using the QIAmp viral RNA kit (Qiagen, Hilden, Germany). Following this, an 83 bp amplicon of the ORF1 region was generated using iTaq™ Universal SYBR^®^ Green One-Step Kit (BioRad) as described in Reference [40]. A standard curve was generated using a serially diluted plasmid (containing the 3′ end of the FCV ORF1) for genome quantitation. The cycling parameters were 50 °C for 20 min, 95 °C for 5 min and 45 cycles of 95 °C for 10 s and 60 °C for 1 min. All reactions were run in duplicate.

### 2.9. Statistical Analysis

Statistical calculations were performed using the GraphPad Prism v.7 software. Data were analyzed using an unpaired *t*-test with Welch’s correction. All error bars depict standard errors of the mean (SEM), and the level of significance are indicated as: *NS*, not significant, *p* > 0.05; * *p* ≤ 0.05; ** *p* ≤ 0.01; *** *p* ≤ 0.001.

## 3. Results

### 3.1. FCV Pro-Pol Expression

We successfully expressed the FCV Pro-Pol polyprotein containing a C-terminal 6-histidine tag in *E. coli* BL21 cells, under the control of the T7 promoter system. From 2 L of the culture, we purified ~3.5 mg of Pro-Pol which appeared at the expected molecular mass (78 kDa) by SDS-PAGE. The presence of the His-tag was confirmed by Western blotting.

### 3.2. RdRp In Vitro Assay

**Figure 1:**
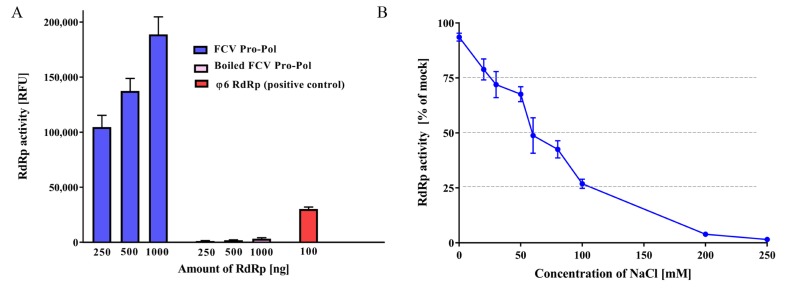
The characterization of FCV RdRp activity. (**A**) Purified recombinant FCV RdRp was used to generate dsRNA from a poly(C) RNA template using rGTP as a substrate (2.5 mM and 0.2 mM final concentration, respectively). Following 1 h of incubation at 30 °C, the reactions were stopped with 10 mM EDTA and dsRNA was quantified using the fluorescent dye PicoGreen. Heat-inactivated FCV RdRp was used as a negative control, and the *Pseudomonas syringae* bacteriophage (φ6) RdRp was used as positive control. (**B**) The effect of NaCl concentration on polymerase activity. Triplicate values from three independent experiments are plotted as the mean ± SEM. The baseline NaCl concentration of the reaction mixture before any additional NaCl was 3 mM. Relative fluorescence units (RFU).

To confirm the RdRp activity of the Pro-Pol dual protein, we tested it using an in vitro fluorescence-based transcription assay, where the dsRNA product was detected with PicoGreen dye [35]. The FCV transcriptional activity increased with increasing concentrations of RdRp (250–1000 ng per reaction) (Figure 1A). Furthermore, a decrease in RdRp activity was observed with increasing NaCl concentration (3–500 mM) (Figure 1B). The RdRp activity was reduced by 50% in the presence of 60 mM NaCl, and completely inhibited at 200 mM (Figure 1B).

### 3.3. Inhibition of RdRp Activity Using NNIs

**Figure 2:**
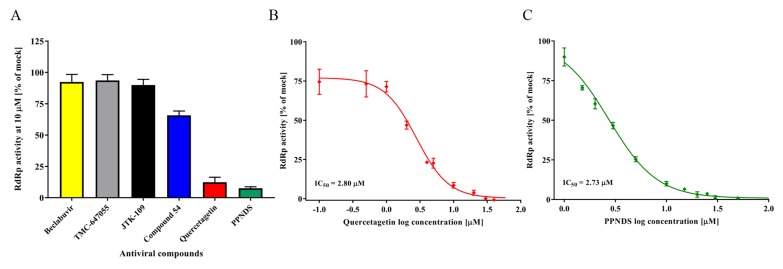
The inhibitory activity of selected non-nucleoside inhibitors against the FCV RdRp. (**A**) The effect of six NNIs was evaluated against FCV RdRp activity at a fixed concentration of 10 µM. Dose-response inhibition trends for PPNDS (**B**) and quercetagetin (**C**) for half maximal inhibitory concentration (IC_50_) determinations. The compounds were tested at concentrations between 0.1 and 100 µM against the FCV RdRp and activity levels were compared to DMSO treatment (vehicle only). Triplicate values from three independent experiments are plotted as the mean ± SEM.

Six NNI compounds (quercetagetin, compound 54, PPNDS, Beclabuvir, TMC-647055, and JTK-109) were tested for FCV RdRp inhibition using the PicoGreen in vitro assay (Table 1). At a fixed concentration of 10 µM, only quercetagetin and PPNDS demonstrated a significant reduction of RdRp activity compared to mock controls (88.3% and 92.6%, respectively) (Figure 2A). Compound 54 slightly reduced the FCV RdRp activity (35%), while all other compounds (Beclabuvir, TMC-647055, and JTK-109) showed a minimal inhibitory effect (≤10%).

Dose-dependent inhibitory response curves (0.1–100 μM) were generated to establish the IC_50_ values for quercetagetin (2.8 μM) and PPNDS (2.7 μM) (Figure 2B,C).

The CC_50_ of each NNI on CRFK cells was determined using the CellTitre-Blue viability assay (Table 1). JTK-109, Beclabuvir, and TMC-647055 demonstrated CC_50_ values of <30 µM, compound 54 showed a CC_50_ value of 55.8 µM, whilst all other NNIs showed values >100 µM (Table 1). In addition, PPNDS and quercetagetin were examined using an FCV plaque reduction assay, with the inhibitory activity calculated after 24 h relative to a mock control (DMSO treatment). At 10 µM, no antiviral activity was observed for both compounds (<10% of plaque formation inhibition) (Table 1).

### 3.4. FCV Protease In Vitro Assay and Test Compounds

**Figure 3:**
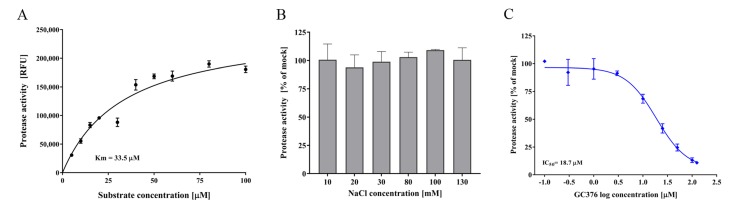
The enzyme kinetics and inhibition of the FCV protease. (**A**) Purified recombinant FCV protease and fluorogenic substrate Dabcyl-FRLE↓ADDG-Edans was used to measure the reaction velocity as a function of the substrate concentration (0 and 100 µM). Following 1 h of incubation at 37 °C, fluorescent signals were measured. Heat-inactivated FCV protease was used as a negative control. (**B**) The effect of NaCl concentration on protease activity. The baseline NaCl concentration of the reaction mixture before any additional NaCl was 3 mM. (**C**) Antiviral activity of the protease inhibitor GC376 against FCV. The half maximal inhibitory concentration (IC_50_) for GC376 against the FCV protease was determined using a FRET assay. Concentrations ranged between 0.1 and 125 µM and FCV protease activity was compared to DMSO treatment (vehicle only). Triplicate values from three independent experiments are shown as the mean ± SEM.

Previous studies have demonstrated that recombinant FCV Pro-Pol exhibits a bifunctional activity of polymerase and protease in vitro [18,55]. Therefore, we also established an in vitro FRET fluorescence-based assay to measure FCV protease activity that cleaves the substrate Dabcyl-FRLE↓ADDG-Edans (corresponding to the LC/VP1 cleavage site) and demonstrated a *K_m_* value of 33.5 µM (Figure 3A). In contrast to FCV RdRp, the protease activity was not inhibited with increasing NaCl concentrations (Figure 3B). The development of the in vitro FRET assay enabled us to test the inhibitory activity of three previously published PIs: GC376 [29], rupintrivir [53] and chymostatin [52]. Of those, only GC376 exhibited an inhibitory effect against the FCV protease in vitro with a 75% inhibition at 50 µM, and further experiments demonstrated an IC_50_ of 18.7 µM (Figure 3C and Table 1).

### 3.5. 2CMC and Nitazoxanide Inhibit FCV Infectivity

**Figure 4:**
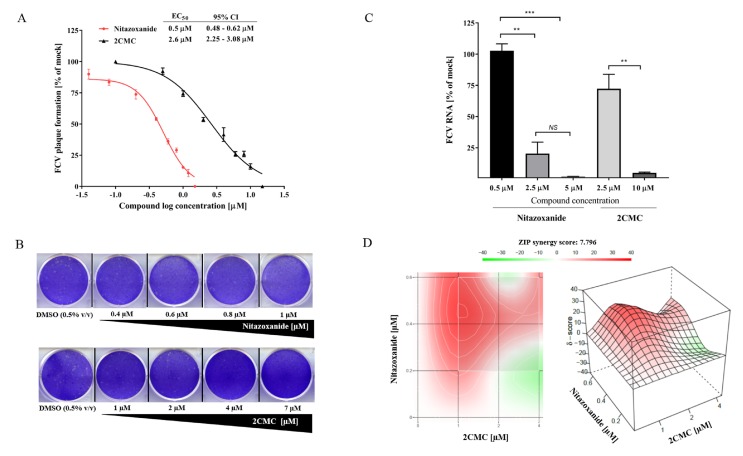
The antiviral activity of 2CMC and nitazoxanide against FCV in the cell culture. (**A**) The EC_50_ values of 2CMC and nitazoxanide against FCV calculated by fitting the dose-response curves from a plaque reduction assay. (**B**) Plaque reduction assay results are shown, with the negative control (vehicle only), nitazoxanide (0.4–1 µM), and 2CMC (1–7 µM). (**C**) Nitazoxanide and 2CMC effects on FCV replication. CRFK cells were infected with FCV (MOI 0.0005) and subsequently treated with different concentrations of both compounds. After 24 h of incubation, FCV RNA copy numbers were determined by RT-qPCR and the relative percentage of RNA copies in relation to mock are plotted. (**D**) The combined inhibitory effects of nitazoxanide (0 to 0.6 µM) and 2CMC (0 to 4 µM) were tested over a range of combinations against FCV in the cell culture using the plaque reduction assay. A dose-response matrix was generated and analyzed for synergy using SynergyFinder. The ZIP mode synergy score is presented as the average of all δ-scores across the dose-response landscape, and the peaks above the plane of 0% synergy in the plot indicate synergism. Nitazoxanide and 2CMC displayed a synergistic antiviral effect against FCV. Data were analyzed using an unpaired t-test. ** *p* < 0.01; *** *p* < 0.001; *NS*, not significant. Duplicate (panels C and D) or triplicate values (panels A and B) from at least two independent experiments are presented, and the mean ± SEM are shown for panels A and C.

Five NA compounds were chosen and tested for their antiviral effects against FCV in the cell culture, including; 2CMC, famciclovir, sofosbuvir, T-705, and 7D2M. All NAs tested have previously shown antiviral effects against several viral families, such as caliciviruses, herpesvirus, paramyxoviruses, orthomyxoviruses, and flaviviruses (Table 1). However, the antiviral efficacy of these compounds against FCV infections has not been evaluated thus far. In addition to these NAs, we also tested the broad-spectrum antimicrobial agent, nitazoxanide, whose mechanism of antiviral action has not been fully elucidated [54]. The dose-response of each compound against FCV was examined using a plaque reduction assay. The compounds 2CMC and nitazoxanide exhibited dose-response inhibition of FCV plaque formation at low micromolar concentrations with EC_50_s of 2.6 µM and 0.6 µM (0.2 µg/mL), respectively (Figure 4A,B). The compound 2CMC demonstrated CC_50_ values of >100 µM, whilst nitazoxanide showed value of 12.7 µM (Table 1), and the therapeutic index values (TI = CC_50_/EC_50_) determined were of >40 and 21.1, respectively.

We also performed RT-qPCR to quantify the FCV RNA levels after antiviral treatment with different concentrations of nitazoxanide or 2CMC. As shown in Figure 4C, a decrease of dose-dependency in FCV RNA levels was observed after 24 h of treatment for both compounds. Nitazoxanide (2.5 µM) resulted in an 80% reduction of FCV RNA levels, whilst 2CMC (10 µM) reduced the RNA levels by 95% compared to the mock-treated cells (Figure 4C).

Sofosbuvir, T-705, and 7D2M showed a modest antiviral activity at 10 µM (<10% inhibition of plaque formation). Famciclovir exhibited minimal FCV antiviral activity at concentrations as high as 50 µM (10% inhibition of plaque formation).

### 3.6. Combinational Treatment with Nitazoxanide and 2CMC Showed Synergistic Antiviral Effects

To determine the synergistic effects of nitazoxanide with 2CMC, we performed plaque reduction assays over several combined concentrations (Figure 4D). The synergistic effect is shown as peaks above the horizontal plane, with ZIP synergy scores varying from 0 to 40. The interaction of both compounds resulted in a moderate synergistic effect (ZIP synergy score of 7.796), with a maximal synergy at concentrations of 0.6:1 μM for nitazoxanide and 2CMC, respectively (Figure 4D).

## 4. Discussion

FCV is a common pathogen of cats and usually associated with acute, mild and self-limiting upper respiratory tract disease, however, more recently highly contagious strains of the virus (FCV-VSD) have been reported in the USA, Europe [11,12,56], and three states of Australia (personal communication, https://au.virbac.com/home/vet-newsletter/main/vet-newsletter/research-update-fcv-vsd.html). With the lack of an effective vaccine and/or antiviral treatment for FCV infection, there is a clear unmet need to identify an effective antiviral agent to improve the management and control of FCV infections.

In the present study, we evaluated 15 different compounds, from four different antiviral classes, using in vitro enzyme- and cell culture-based assays, 13 of which have not previously been evaluated against this virus.

We identified the NA 2CMC (EC_50_ = 2.5 μM) and the broad spectrum antimicrobial compound nitazoxanide (EC_50_ = 0.6 μM or 0.2 µg/mL) as potent inhibitors of FCV replication (Figure 4A and Table 1). An NA originally designed for use against HCV, 2CMC is a promising calicivirus antiviral and has previously been tested against human and murine norovirus, with similar results to our current study [57,58,59]. Using in vitro assays, Jin et al. [59] showed that tri-phosphorylated 2CMC inhibited human and murine norovirus RdRp activity with IC_50_s of 2.4 μM and 1.4 μM, respectively. In the same study, 2CMC was tested against the human GI.1 norovirus replicon in the cell culture and demonstrated an EC_50_ of 8.2 μM. In related studies, 2CMC was also shown to inhibit the murine norovirus (EC_50_ ~2 μM) and the human norovirus replicon (EC_50_ ~18 μM) using cell culture-based assays [47,57]. Recently, using a B-cell culture system, 2CMC also effectively inhibited the human norovirus with an EC_50_ of 0.3 μM [60]. Therefore, our results are consistent with the inhibitory data obtained against other caliciviruses as reported in the above cited studies.

Valopicitabine, the prodrug form of 2CMC, was used in pre-clinical studies to treat HCV infections, however, after dose-related gastrointestinal adverse events, the drug has been placed on clinical hold by the US Food and Drug Administration (FDA). Although promising results were obtained here and in other studies [58,60], concerns over adverse side-effects may limit its future clinical use to treat calicivirus infections.

Nitazoxanide is a broad-spectrum antimicrobial compound with activity against anaerobic bacteria, protozoa, and viruses [54]. It is an FDA-approved drug licensed for gastroenteritis caused by the parasites *Cryptosporidium parvum* and *Giardia intestinalis* [61,62]. In cell cultures, nitazoxanide has been evaluated against several viruses, showing inhibition in the replication of rotavirus (EC_50_ 0.5 µg/mL), adenovirus (EC_50_ 0.2 µg/mL), canine coronavirus (EC_50_ 1 µg/mL), influenza viruses (EC_50_ 0.2–1.5 µg/mL), among others [54,63]. In the present study, nitazoxanide demonstrated an EC_50_ of 0.2 µg/mL (0.6 μM) against FCV, which is within the range of values found when tested on other viruses. Recently, the drug was reported to inhibit GI norovirus replicon replication at 5 μg/mL, and cleared the replicon from the host cells, but was ineffective against murine norovirus [64].

Nitazoxanide has been commercialized in Latin American countries and India to treat a broad spectrum of intestinal parasitic infections and is currently in clinical trials to treat norovirus gastroenteritis [54,63]. For example, a large randomized, double-blind, placebo-controlled clinical trial is being conducted using nitazoxanide to treat acute gastroenteritis mainly caused by *Cryptosporidium parvum*, norovirus, and rotavirus in hospitalized aboriginal children in the Northern Territory, Australia [65]. There is also some published anecdotal evidence that this drug works on norovirus in a small number of case studies [66,67].

In the veterinary field, small animals such as cats and dogs have received nitazoxanide to treat intestinal parasites. Gookin et al. [68] demonstrated the successful use of nitazoxanide in eliminating the shedding of *Tritrichomonas foetus*, a cause of chronic diarrhea in cats. In another study, the successful administration of nitazoxanide to treat giardiasis and cryptosporidiosis in dogs was demonstrated [69]. Given that nitazoxanide displayed a potent inhibition against FCV and is already used in a clinical setting for feline infections, our data illustrate that nitazoxanide could be repurposed for the treatment of FCV infections. However, considering the narrow in vitro therapeutic index of nitazoxanide, and its side-effects (diarrhea and vomiting) observed in cats after nitazoxanide administration [68], concerns about the effective dose in vivo should be addressed.

The combination of antiviral compounds with additive or synergistic effects is a strategy to improve drug efficacy, reduce antiviral toxicity, and limit the development of viral resistance. Here, we demonstrated that the combination of nitazoxanide and 2CMC in cell cultures had a synergistic inhibitory effect against FCV, with an average delta score of 7.79 (Figure 4D). As nitazoxanide showed cytotoxicity on CRFK cells at a relatively low concentration (CC_50_ = 12.7 μM), the synergistic effect resulted from the combination with 2CMC (CC_50_ > 100 μM) could be useful in limiting its cytotoxic effects by reducing the effective concentration of nitazoxanide, and overall improving the efficacy of the combination treatment.

In the present study, we have expressed the recombinant FCV Pro-Pol with high yields of active protein. Previous studies have demonstrated that the fusion protein is stably expressed in FCV-infected cells and is the primary and active form of the protein, which maintains both protease and polymerase activity [18,70]. As previously shown by Wei et al. [18], we also demonstrated that high concentrations of NaCl (100 mM) caused a reduction in the RdRp activity (Figure 1B), however, no effect in the protease activity was observed at this concentration (Figure 3B).

Of the six NNI compounds tested in the current study, PPNDS and quercetagetin showed an inhibition of FCV RdRp activity with IC_50_ values in the low micromolar range (Figure 2 and Table 1). In previous studies, PPNDS demonstrated potent inhibition of RdRp activity against viruses from three calicivirus genera, *Norovirus*, *Sapovirus*, and *Lagovirus*, with IC_50_ values between 0.1 and 2.3 µM [33,42,71]. However, due to cell permeability issues limiting bioavailability and antiviral efficacy in cell cultures, PPNDS is not considered a potential antiviral drug candidate [72,73]. While in the current study quercetagetin displayed an IC_50_ of 2.8 μM in polymerase assays, it did not inhibit FCV plaque formation and therefore is not a suitable FCV antiviral. Quercetagetin, a natural flavonoid compound, was first reported as a potent inhibitor of HCV replication in vitro [74]. The compound demonstrated a potent RdRp inhibition against different HCV genotypes, with IC_50_s between 2.8 and 6.1 µM, but was less potent in cell cultures against the infectious virus (EC_50_ 40.2 µM ± 17.7) [74]. Quercetagetin also showed a moderate inhibitory activity against the chikungunya replicon, with an IC_50_ of 43.5 µM [75].

In addition to the polymerase inhibition assay, using the purified FCV Pro-Pol, we also described a FRET protease assay for high throughput screening of FCV protease inhibitors. As with viral polymerases, proteases play a crucial role in the viral replication cycle and are attractive targets for antiviral development. Several viral PIs are currently approved or under development to treat pathogenic viruses such as HIV, HCV, and the SARS coronavirus [76,77]. GC376 is under development for feline coronavirus infection (feline infectious peritonitis) [78]. Using the FRET-based assay, we tested three previously published PIs, with only GC376 demonstrating a moderate inhibition against the protease (IC_50_ of 18.7 µM) (Figure 3C). This compound has previously shown a potent inhibition against the proteases of norovirus, coronaviruses, and picornaviruses, with IC_50_s ranging from 0.20 to 4.35 µM [29]. However, against FCV in cell-based assays, an EC_50_ value of 35 µM was obtained [29], similar to the value obtained in our study. The PIs rupintrivir and chymostatin have previously demonstrated an inhibition of the human norovirus protease (genogroup I and II) in FRET-based assays, with IC_50_ values of <1 µM and 5–10 µM, respectively [29,52]. However, no inhibitory effect was observed for either PI against FCV protease in this study.

Among the NAs tested, famciclovir is used for the treatment of feline herpesvirus (FHV)-associated clinical disease [79]. This drug is also commercially used as an FCV and FHV treatment. We tested famciclovir at concentrations up to 50 µM using the cell culture plaque reduction assay with no antiviral effect observed. Our data show that the compound is ineffective at inhibiting virus replication and thus is a poor therapeutic option for the treatment of FCV infections.

FCV is a highly infectious respiratory pathogen of cats with a global distribution, and more recently FCV-VSD associated high-mortality outbreaks have been reported. Despite the availability of a vaccine, the high diversity of the FCV genome plays a key role in vaccine failure and is also the basis for the emergence of virulent strains. In addition, there are currently no approved antivirals to treat the disease. Here, we report the establishment of two in vitro assays that allow for the identification of novel inhibitors of the FCV polymerase and protease. The present findings have implications for the development of FCV antivirals, providing a basis to design and select drugs which may be used in the veterinary clinic. Using the in vitro assays, we identified quercetagetin and PPNDS as potent RdRp inhibitors, and we also demonstrated a moderate inhibition of protease activity by GC376. Finally, we reported the identification of two compounds (nitazoxanide and 2CMC) with antiviral activity against FCV in cell culture at low micromolar concentrations with a potential combinational therapeutic utility to treat FCV-infected cats.

## Figures and Tables

**Table 1:** The compounds used in this study to test antiviral activity against FCV.

Compound	Molecular Mass (g/mol)	Stage of Antiviral Development	Original Target Viral Family	Antiviral Class	Cell Viability CC_50_ (µM)	Plaque Reduction Assay EC_50_ (µM)	RdRp Activity [% of Mock at 10 µM] (IC_50_ µM)	In vitro Protease IC_50_ (µM)	Reference
Quercetagetin	318.2	Research	*Herpesviridae*	NNI	>100	>10	11.7 (2.8)	ND	Cotin et al. [41]
PPNDS	694.3	Research	*Caliciviridae*	NNI	>100	>10	7.4 (2.7)	ND	Tarantino et al. [42]
Compound 54	485.5	Research	*Caliciviridae*	NNI	55.8	ND	65.7	ND	Ferla et al. [43]
Beclabuvir	659.8	Phase 2	*Flaviviridae*	NNI	27.5	ND	92.2	ND	Gentles et al. [44]
TMC-647055	606.7	Phase 2	*Flaviviridae*	NNI	27.1	ND	93.5	ND	Devogelaere et al. [45]
JTK-109	638.1	Phase 2	*Flaviviridae*	NNI	11.9	ND	89.8	ND	Hirashima et al. [46]
2CMC	257.2	Pre-clinical	*Flaviviridae*	NA	>100	2.6	ND	ND	Rocha-Pereira et al. [47]
Sofosbuvir	529.5	Approved	*Flaviviridae*	NA	>100	>10	ND	ND	Lam et al. [48]
T-705	157.1	Phase 3	*Orthomyxoviridae*	NA	>100	>10	ND	ND	Furuta et al. [49]
7D2M	280.3	Research	*Flaviviridae*	NA	>100	>10	ND	ND	Olsen et al. [50]
Famciclovir	321.3	Approved	*Herpesviridae*	NA	>100	>50	ND	ND	Boyd et al. [51]
GC376	507.5	Research	Broad spectrum	PI	>100	>10	ND	18.7	Kim et al. [29]
Chymostatin	607.7	Research	*Caliciviridae*	PI	ND	ND	ND	>50	Chang et al. [52]
Rupintrivir	598.7	Research	*Picornaviridae*	PI	>100	ND	ND	>50	Dragovich et al. [53]
Nitazoxanide	307.3	Approved	Broad spectrum	Unknown	12.7	0.6	>10	>10	Rossignol [54]
